# Supplementary material for: Renal function impairment in cervical cancer patients treated with cisplatin-based chemoradiation: A review of medical records in a Zimbabwean outpatient department
Source: PLoS One. 2021 Feb 24;16(2):e0245383. doi: 10.1371/journal.pone.0245383 (PMC7904141; doi:10.1371/journal.pone.0245383)
Supplement: Attachment — Submitted filename: Response letter.pdf [file pone.0245383.s002.pdf]

**UNIVERSITY OF ZIMBABWE**
**FACULTY OF MEDICINE AND HEALTH SCIENCES**
**DEPARTMENT OF PHARMACY AND PHARMACEUTICAL SCIENCES**

14 December, 2020

**RE: Renal function impairment in cervical cancer patients treated with cisplatin-based chemoradiation in a Zimbabwean clinical setting (PONE-D-20-14840).**

Dear editors,

Thank you for your positive and constructive feedback for the manuscript. The manuscript has been revised in accordance with your recommendations. Our responses for each of the concern raised by the reviewers are provided overleaf (in red). We are also submitting two versions of the manuscript; one with the highlighted changes and an unmarked version.

Yours's Faithfully

Pinky M.C Manyau

***BPharm (Hons), MSc***

1. Please ensure that your manuscript meets PLOS ONE's style requirements, including those for file naming. The PLOS ONE style templates can be found at https://journals.plos.org/plosone/s/file?id=wjVg/PLOSOne_formatting_sample_main_body.pdf and https://journals.plos.org/plosone/s/file?id=ba62/PLOSOne_formatting_sample_title_authors_affiliations.pdf

The manuscript has been revised as follows:

- Line numbers have been inserted;
- Level 1 and 2 headings have been inserted for major and minor headings respectively;
- "Figure 1" has been converted to .tif format.

2. In the ethics statement in the manuscript and in the online submission form, please provide additional information about the patient records used in your retrospective study. Specifically, please ensure that you have discussed whether all data were fully anonymized before you accessed them and/or whether the IRB or ethics committee waived the requirement for informed consent. If patients provided informed written consent to have data from their medical records used in research, please include this information."

The ethics statement has been revised to:
"All data were fully anonymized before they were accessed. Ethics approvals were obtained from the Joint Research and Ethics Committee for University of Zimbabwe College of Health Sciences and Parirenyatwa Group of Hospitals (JREC#:315/19) before commencement of the study".

3. To comply with PLOS ONE submission guidelines, in your Methods section, please provide additional information regarding your statistical analyses. For more information on PLOS ONE's expectations for statistical reporting, please see https://journals.plos.org/plosone/s/submission-guidelines.#loc-statistical-reporting."

More detail has been provided for the following:

- Descriptive statistics;
- Baseline comparisons between HIV-infected and -uninfected patients;
- All p-values have been reported to 3 decimal places;
- Use and presentation of odds ratios obtained from regression analysis

Table 3 which presents results for regression analysis has also been amended. The 95% confidence interval which was initially in the same column as the p-value has been placed in the same column as the point estimate.

4. Please include a caption for figure 1.

The caption for figure 1 has been inserted after the paragraph where the figure is first cited (line 161-163).

5. Please include captions for your Supporting Information files at the end of your manuscript, and update any in-text citations to match accordingly. Please see our Supporting

Information guidelines for more information: http://journals.plos.org/plosone/s/supporting-information.

The heading "Supporting Information" along with the description ("Data")

Reviewers' comments:

Reviewer's Responses to Questions
**Comments to the Author**

1. Is the manuscript technically sound, and do the data support the conclusions?

The manuscript must describe a technically sound piece of scientific research with data that supports the conclusions. Experiments must have been conducted rigorously, with appropriate controls, replication, and sample sizes. The conclusions must be drawn appropriately based on the data presented.
Reviewer #1: Yes

---

2. Has the statistical analysis been performed appropriately and rigorously?
Reviewer #1: Yes

---

3. Have the authors made all data underlying the findings in their manuscript fully available?

The PLOS Data policy requires authors to make all data underlying the findings described in their manuscript fully available without restriction, with rare exception (please refer to the Data Availability Statement in the manuscript PDF file). The data should be provided as part of the manuscript or its supporting information, or deposited to a public repository. For example, in addition to summary statistics, the data points behind means, medians and variance measures should be available. If there are restrictions on publicly sharing data—e.g. participant privacy or use of data from a third party—those must be specified.
Reviewer #1: Yes

---

4. Is the manuscript presented in an intelligible fashion and written in standard English?

PLOS ONE does not copyedit accepted manuscripts, so the language in submitted articles must be clear, correct, and unambiguous. Any typographical or grammatical errors should be corrected at revision, so please note any specific errors here.
Reviewer #1: Yes

---

5. Review Comments to the Author

Please use the space provided to explain your answers to the questions above. You may also include additional comments for the author, including concerns about dual publication, research ethics, or publication ethics. (Please upload your review as an attachment if it exceeds 20,000 characters)

Reviewer #1: This paper studied the relationship between HIV-infection and increased renal impairment in ICC patients in Zimbabwe. They found the prevalence of renal impairment in the study is higher than patients in other counties and gave some suggestions for the

treatment. This study is well-designed and the results are reliable. However, there are some issues exist.

1. The first paragraph needs a subtitle.
The first paragraph has been titled “Background”

2. The subtitle "Changes in renal function" should be changed to a more suitable one to reflect the findings you described.

The section reports the distribution of adverse renal events (lowest eGFR <60ml/min, percent reductions in eGFR, and grading of adverse renal events), hence the subtitle has been changed to “Adverse renal events”.

3. Both "GFR" and "eGFR" are used in this manuscript. What is the difference between them?

We reported estimated GFR based on creatinine clearance hence the term eGFR has been adopted for consistent use.

4. All 3 panels in Figure 1 lack the names y-axis. Also, it is better to add P values to all 3 panels.

The y-axis was labelled “% Change”, and the respective p=values have been embedded in the figure.
